# Amination of the Gd@C_82_ endohedral fullerene: tunable substitution effect on quantum coherence behaviors†

**DOI:** 10.1039/d0sc02182b

**Published:** 2020-05-28

**Authors:** Zheng Liu, Huan Huang, Ye-Xin Wang, Bo-Wei Dong, Bao-Yun Sun, Shang-Da Jiang, Song Gao

**Affiliations:** Beijing National Laboratory of Molecular Science, Beijing Key Laboratory of Magnetoelectric Materials and Devices, College of Chemistry and Molecular Engineering, Peking University Beijing 100871 China jiangsd@pku.edu.cn gaosong@pku.edu.cn; School of Chemistry and Chemical Engineering, South China University of Technology Guangzhou 510640 China; Beijing Academy of Quantum Information Sciences West Bld. #3, No. 10 Xibeiwang East Rd., Haidian District Beijing 100193 P. R. China; CAS Key Lab for Biomedical Effects of Nanomaterials and Nanosafety, Institute of High Energy Physics, Chinese Academy of Sciences Beijing 100049 China sunby@ihep.ac.cn

## Abstract

The core–shell structure of endohedral fullerene-based anisotropic magnetic molecules of high spin with long coherence time could help scale up quantum systems. In this research, by amination of Gd@C_82_ with morpholine, three derivatives are functionalized with 5, 7 and 9 morpholine groups providing an interesting model to investigate the relationship between the quantum coherence and the spin environment. The original radical located on the carbon cage is successfully quenched, affording a quantum phase memory times (*T*_M_) over 5 μs at 5 K. By increasing the number of substitution groups, spin–lattice relaxation times (*T*_1_) also show significant enhancement due to the interaction variation between the molecules and the environments. We found that the *T*_M_ of the three molecules show no obvious difference below 10 K, while they are limited by *T*_1_ at higher temperatures. In this work, the variable functional groups are able to tune both *T*_1_ and *T*_M_, offering the possibility for application of high-spin magnetic molecules in the field of quantum information processing.

## Abstract 2

Amination of the endohedral fullerene Gd@C82 exhibits enhanced quantum phase memory time and tunable substitution effect for quantum information processing.

## Graphical abstract

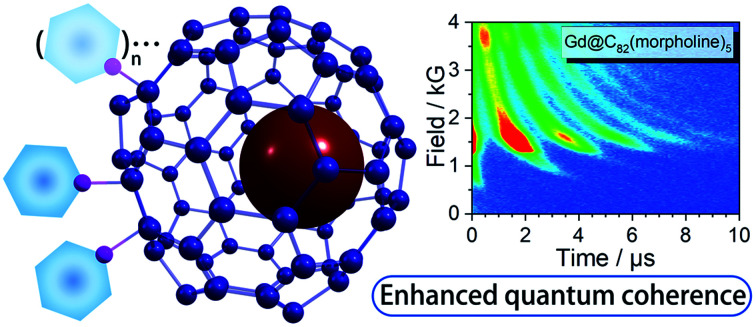

## Introduction

Endohedral fullerenes with encapsulated metal atoms, ions or clusters in the carbon cage have shown lots of distinctive properties compared to the hollow ones due to the electron transfer between the inner core and the outer shell. Plenty of applications of endohedral fullerenes have been investigated including in chemistry,^1^ electronics^2^ and reactions.^3^ Additionally, paramagnetic endohedral fullerenes containing well protected unpaired electron spins show unique characteristics compared to radicals and complexes. The various paramagnetic properties can be applied in dynamic nuclear polarization (DNP),^4^ magnetic resonance imaging (MRI) contrast agents,^5,6^ molecular magnets^7^ and biomedicines.^8,9^

Recently, quantum information processing (QIP) has been proved to be able to provide efficient solutions in cryptography^10,11^ and database searching.^12^ Molecular spins, being much more versatile as building blocks, are becoming the competitive one in the area of QIP.^13^ The high spin state of molecular magnet-based “qudits” fits the requirements for Grover's algorithm proposed by Leuenberger and Loss.^14^ This proposal requires the spin system to possess non-equidistant energy levels and long enough quantum phase memory time. The design of magnetic coordination complexes will give us a chance in fine controlling the quantum coherence and building quantum gates.^15^ However, there is a significant contradiction between these conditions since the quantum coherence of an anisotropic high-spin system can be easily destroyed due to its strong coupling to the environment. One possible protocol to solve this problem is to employ rare earth ions of high spin ground state with small anisotropy. The core–shell structures of endohedral fullerenes could fulfill the requirements in QIP,^16,17^ and show even better properties compared to other molecule-based qubits. Various paramagnetic endohedral fullerenes show some particular phenomena including the qubit crossover phenomenon^18^ and diverse Rabi cycles.^19^

However, most investigations of paramagnetic endohedral fullerenes are limited to pristine molecules. Functionalization of fullerenes can precisely tune the spin environment,^20,21^ helping us understand the spin dynamics. Gd@C_82_ is a well-known endohedral fullerene with the paramagnetic metal ion Gd^3+^ of *S* = 7/2 for the inner core and C_82_^3−^ acting as a radical located at the outer shell. The antiferromagnetic coupling between Gd^3+^ and the radical makes an *S* = 3 ground state.^22^ Since the electron cloud of the radical is largely scattered among the outer shell with lack of protection, the properties of the spin system are largely influenced by the environment. This phenomenon is a disadvantage because the uncontrollable interactions will largely reduce the performance of endohedral fullerenes in QIP. The distribution of the electron cloud could enhance the influence of the environment and largely reduce the quantum coherence time. Herein, to overcome this disadvantage, we successfully quench the unwanted shell-radical by amination of the fullerene cage, obtaining three derivatives Gd@C_82_(morpholine)_*n*_ (marked as **Gd-n**, *n* = 5, 7 and 9).

## Results & discussion

In our previous studies,^23,24^ the amination of Gd@C_82_ with morpholine and *N*-fluorobenzenesulfonimide (NFSI) resulted in the selective formation of three derivatives, **Gd-5**, **Gd-7** and **Gd-9**. The structure of **Gd-7** has been confirmed by X-ray crystallographic analysis in the previous report. The evidence including UV-Vis-NIR spectroscopy, electrochemical analysis and continuous wave electron paramagnetic resonance (cw-EPR) demonstrated that this series of **Gd-n** derivative compounds are functionalized with an odd number of morpholine groups. Different from the original endohedral fullerene Gd@C_82_, the absence of the radical leads to a relatively independent environment with controllable differences. The comparison of this series of molecules provides a very interesting model to investigate the quantum coherence behavior with a tunable environment influence. In this work, we move forward to the different behaviors of the spin–lattice relaxation time (*T*_1_) and quantum phase memory time (*T*_M_).

To evidence the quenching of the radical, the magnetizations of **Gd-7** were measured at 2 K up to 5 T on a magnetometer as shown in Fig. S2-1.† The field-dependent magnetization curve and the saturation value fit best to the *S* = 7/2 model of the Brillouin function. These results confirm the disappearance of the radical in **Gd-n** molecules (ESI†).

### cw-EPR characterization

In the previous research, the spin system of the Gd@C_82_ endohedral fullerene was considered as the combination of one high spin Gd^3+^ center of *S* = 7/2 and one radical of *S* = 1/2 delocalized on the carbon cage. The two spins were antiferromagnetically coupled as an *S* = 3 system according to high-frequency EPR measurements.^22^ However, the radical at the carbon cage reduces the protection of the spin system efficiently, which can be certified from measurements under different conditions. Such influence will also lead to poor quantum coherence properties.

**Fig. 1:**
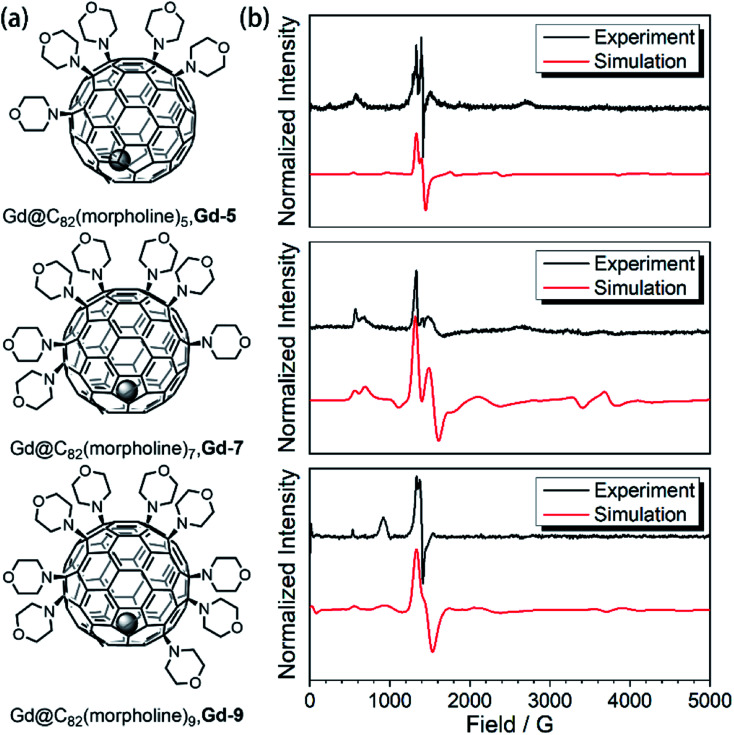
(a) Aminated derivatives of Gd@C_82_ with different numbers of morpholine groups. (b) The cw-EPR measurements for the **Gd-n** derivatives (black) and the best simulation results (red).

With the shell radical quenched, the Gd^3+^ ion is the only electron spin carrier in the molecules. Even though the first order orbit angular momentum of the Gd^3+^ ion is fully quenched, a slight magnetic anisotropy can still be observed due to the second order spin–orbit coupling in the existence of broken environmental symmetry. Gd@C_82_ and the three derivative compounds were resolved in a d_8_-toluene solution with relatively low concentrations from 5 K to 150 K as reported in a previous study. The X-band cw-EPR offers us a way to probe the crystal field effect with various chemical modifications on the fullerene cage. Therefore, the spin Hamiltonian of **Gd-n** is considered as1
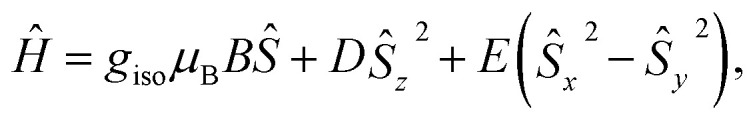
where the first term describes the Zeeman effect and the latter two represent the second-rank zero-field splitting (ZFS) of rhombic symmetry. As no significant hyperfine interaction was evidenced in the cw-EPR spectra (^1^H, ^2^D, ^14^N, ^155^Gd or ^157^Gd), the hyperfine interaction and nuclei quadrupole terms are not included in the Hamiltonian. The simulations of the experimental curves of the derivatives at 20 K (Fig. 1) provided three sets of parameters as the best fitting result for each spectrum, as listed in Table 1. Compared to the Gd^3+^ center of the original Gd@C_82_ molecule, ZFS parameters are different for those of the three derivatives, indicating that the electron spins have rearranged, suggesting differences in quantum coherence behaviors.

**Table 1:** The spin Hamiltonian parameters for the three derivative compounds

Compound	*S*	*g*	*D*	*E*
Gd^3+^ of Gd@C_82_ (ref. 22)	7/2	(2.0090, 2.0100, 1.9775)	0.2575 cm^−1^	0.0070 cm^−1^
Gd@C_82_(morpholine)_5_ (ref. 24)	7/2	1.96	0.3069 cm^−1^	0.0280 cm^−1^
Gd@C_82_(morpholine)_7_ (ref. 24)	7/2	1.98	0.2175 cm^−1^	0.0217 cm^−1^
Gd@C_82_(morpholine)_9_ (ref. 24)	7/2	1.99	0.2542 cm^−1^	0.0252 cm^−1^

### Quantum coherence properties

The aforementioned results illustrated that the Gd^3+^ electronic structures can be varied by chemical modification on the outer shell. The protocol for the quantum searching algorithm with molecular magnets proposed by Leuenberger and Loss requires non-equidistant energy levels. The present approach of modifying electronic structures of paramagnetic ions makes the employment of **Gd-n** as a qudit (*d* = 8) possible.^25,26^ Now we move to study how the substitution groups affect the spin dynamics behaviors. According to the simulations of the cw-EPR spectra of the three derivatives, the D values (0.2–0.3 cm^−1^) are comparable to the microwave frequency (∼9.3 GHz) used in an X-band EPR spectrometer. Only several transitions were observed in the experiments. In the echo-detected field swept (EDFS) experiments, the standard Hahn echo pulse sequence (π/2–*τ*–π–*τ*–echo with π/2- and π-pulses of 16 and 32 ns, respectively) was used to record the electron spin echo intensities from 1 to 4000 G for **Gd-n** at 5 K.

**Fig. 2:**
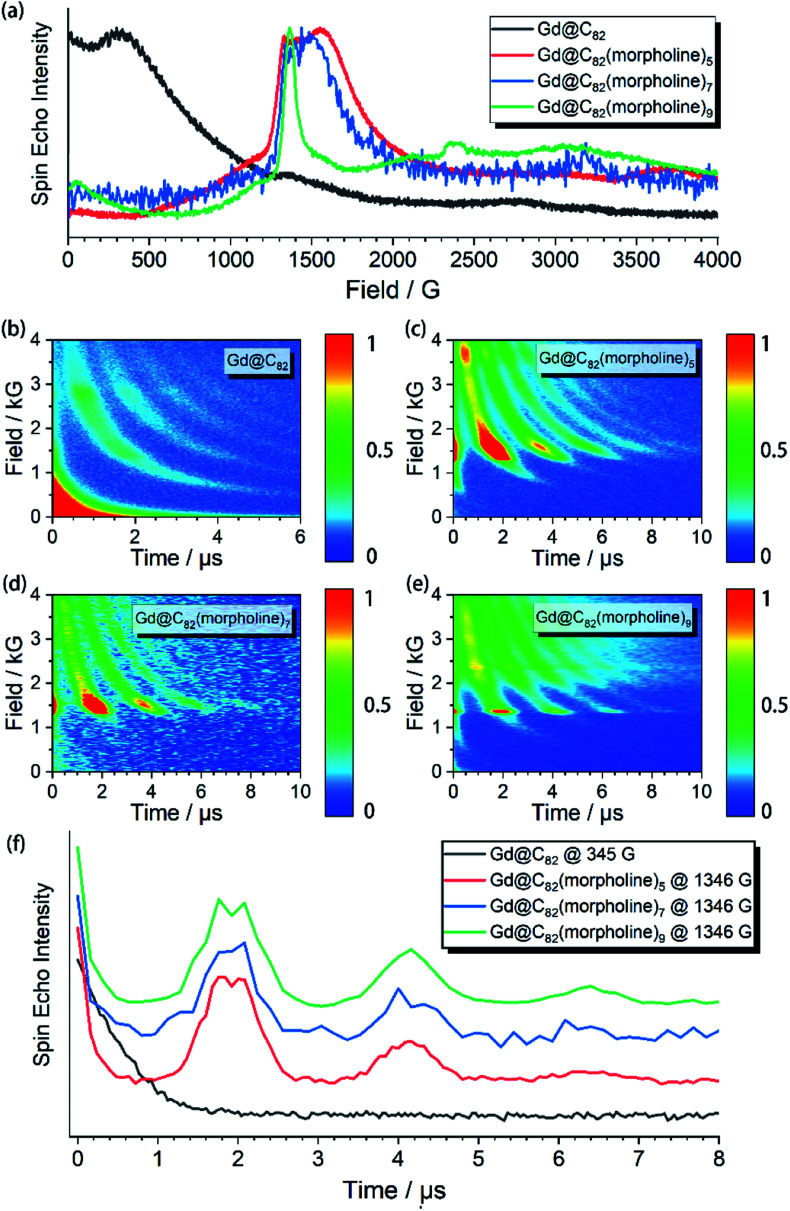
(a) Echo-detected field swept spectra of Gd@C_82_ and the three derivatives **Gd-n** with normalized echo intensities; (b)–(e) the spin echo decay at different external applied magnetic fields (*B*_0_) of each compound; (f) decay of the Hahn-echo intensity as a function of delay time for Gd@C_82_ and the three derivatives **Gd-n** at specific *B*_0_.

**Fig. 3:**
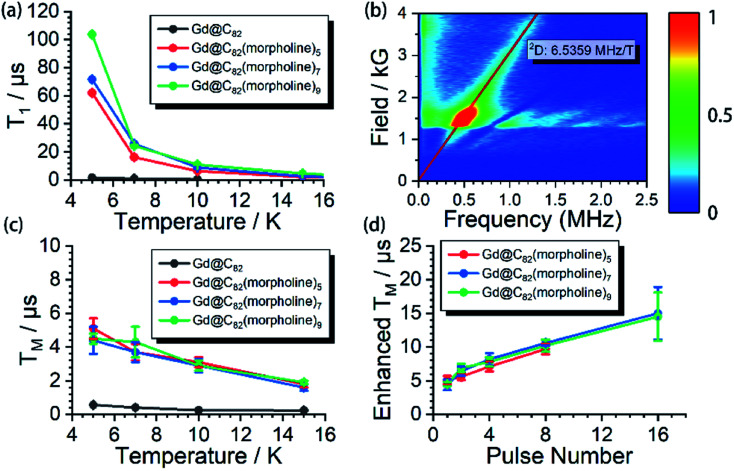
(a) Spin–lattice relaxation time (*T*_1_) of Gd@C_82_ and the three derivatives **Gd-n**. (b) 2p-ESEEM spectrum at different external applied magnetic fields for **Gd-5**. The red line represents the simulation with the Lamour frequency of the ^2^D nuclear spin. (c) Spin–lattice relaxation time (*T*_1_) of Gd@C_82_ and the three derivatives **Gd-n**. (d) Dynamic decoupling results for the three derivatives **Gd-n**.

As shown in Fig. 2a, several transitions at different magnetic fields can be observed. In the measurements of electron spin *T*_M_ at 5 K, one can observe that the *T*_M_ values strongly depend on the external magnetic field (*B*_0_) as shown in Fig. 2b–e. It can be clearly seen that Gd@C_82_ shows a different pattern compared to the three derivatives. These patterns are mainly influenced by the electronic structures and the surrounding environments. Peaks in the vertical cuts indicate the resonance magnetic fields, representing the transitions which can be calculated from the spin Hamiltonian parameters. In a certain applied magnetic field, the decay of the spin echo is modulated at frequencies that depend on the field, which is attributed to the electron-spin echo envelope modulation (ESEEM) effect. The horizontal cuts of all the compounds show the modulations shown in Fig. 2f. By choosing the same *B*_0_, the spin echoes oscillate at the same frequency, which represents the same influences from the surrounding nuclear spins. The Fourier transformations of the pattern of **Gd-5** are shown in Fig. 3b. The slope of 6.59 MHz T^−1^ indicates that the electron spin is coupled to the deuterium nuclei from the solvent (*γ*_D_ = 6.5359 MHz T^−1^).

The temperature dependence of electron spin quantum coherence properties was investigated at the *B*_0_ field that gave the biggest transition probabilities and therefore the strongest signals. Based on the determined spin Hamiltonian parameters, the transition assignment at various magnetic fields can be easily calculated. For Gd@C_82_, the transition between |−1/2〉 and |+1/2〉 at 345 G was selected, and for the three derivative compounds, the transitions between |+1/2〉 and |+3/2〉 at around 1400 G were selected.

### Quantum coherence behaviors

The spin echo can be observed up to 40 K for the three derivatives. *T*_1_ and *T*_M_ at different temperatures are shown in Fig. 3. It can be clearly seen that both *T*_1_ and *T*_M_ show clear response to the environment temperature. The *T*_1_ of Gd@C_82_ is two orders of magnitude smaller than that of **Gd-n**. The delocalized radical efficiently enhances the interaction between the electron spin and the environment. As a result, the thermally activated spin–lattice relaxation is therefore largely accelerated *via* the strong exchange of phonons between the cage and the environment. However in contrast, the phonon exchange between the inner magnetic ion and the environment is much less efficient, affording longer spin–lattice relaxation times in the derivatives due to the isolated spin environment. At a lower temperature of 5 K, *T*_1_ values of all **Gd-n** derivatives are around 100 μs and decrease to less than 1 μs upon warming to 40 K. Within the three analogs, *T*_1_ increases obviously with more substitution groups. This is probably due to the tougher restrictions imposed on the molecular tumbling. Fullerenes with more substitutions would have slower molecular tumbling rates,^27^ which influence the rate of the vertical relaxation.^28^ In our experiments, this effect of having more substitution groups appears to slow down the spin–lattice relaxation.

The quantum coherence properties of these endohedral fullerenes are also distinguishable. Due to the strong coupling with the environment arising from the radical of the outer shell, the *T*_M_ of only 1.4 μs was observed for Gd@C_82_ at 5 K and dropped below 1 μs upon warming. Nevertheless, the *T*_M_ of the high spin derivatives with quenched radicals can be enhanced to more than 5 μs, which are even better than those of VO^2+^ (*S* = 1/2) and Cr^3+^ (*S* = 3/2) based low spin molecules.^29–31^ The temperature dependence of the quantum phase memory time shows a different trend compared to the spin–lattice relaxation time. Upon warming from 5 K to 10 K, *T*_M_ decreases slightly for the three analogs. Different from the strong relation between *T*_1_ and temperature, the decoherence of the electron spin superposition state is caused by additional sources, including the dipolar interaction between the investigated electron spin and the environmental nuclear or electron spins.^32,33^ The molecular concentrations in these experiments were low enough to consider that the electron spin dipolar–dipolar interaction was a minor effect that resulted in the decoherence path here.^34^ The aforementioned ESEEM effects indicate that the electron spin of Gd^3+^ is effectively coupled to the environment nuclei. Based on these analyses, we confirm that the hyperfine coupling of the nuclear spins dominates the decoherence of the electron spin superposition states in the three derivatives at relatively low temperatures from 5 to 10 K. By warming up the spin system above 10 K, *T*_M_ decreases with the same trend as *T*_1_. The *T*_1_ of each molecule is around twice of *T*_M_, and even closer at higher temperatures. Therefore, the thermal process becomes the main decoherence path above 10 K. It is also interesting to compare the quantum coherence time of the three derivatives. The molecule with the least number of substituted morpholine groups shows the longest phase memory time at 5 K. While upon warming, the *T*_M_ of **Gd-5** drops more rapidly than that of the other two. This is mainly due to the *T*_1_ limitation, indicating that the molecules with a longer spin–lattice relaxation time are less limited at relatively higher temperatures.

### Dynamic decoupling

The nuclear spins around the electron spin can generate the local Overhauser field which oscillates at Larmor frequencies. As discussed previously, these nuclear spins acted as the major decoherence path in our experiment at relatively low temperatures. This unwanted environment effect can be decoupled by employing a train of refocusing pulses before the detection of the spin echo.^35^ Here, the CPMG-*n* pulse sequence was applied to the three derivatives at 5 K, where *n* represents the number of the inversion pulses. This dynamic decoupling pulse sequence exhibits very efficient enhancement of *T*_M_. By applying up to 16 inversion pulses, the phase memory times were successfully extended to nearly 20 μs, which is 4 fold higher compared to the original phase memory times. These dynamic decoupling experiments suggest that by employing refocusing pulses, the interactions between electrons and surrounding nuclear spins are successfully decoupled. It is also worth noting that *T*_M_ can be further extended with more than 16 refocusing pulses; however, the detection is limited by the weak signals.

## Conclusions

In summary, the quantum coherence behaviors of the three derivative compounds of the endohedral fullerene Gd@C_82_ with an odd number of morpholine groups, Gd@C_82_(morpholine)_*n*_ (*n* = 5, 7 and 9), have been investigated using pulse-EPR. By quenching the radical located at the carbon cage, the electronic structures become largely different from those of the original Gd@C_82_. The quantum phase memory times (*T*_M_) can be enhanced to more than 5 μs at 5 K which are even better compared to that of low spin molecules. While, with the increasing number of substitution groups, spin–lattice relaxation times (*T*_1_) also show significant enhancement due to the interaction variation between the molecules and environment. Besides, it is also worth noting that *T*_M_ show no obvious difference below 10 K, while they are limited by *T*_1_ at higher temperatures. Gd^3+^ based endohedral fullerenes have been widely used in interesting studies including medical development and quantum information processing, and precisely controlling the quantum coherence properties are required for better applications. In this work, **Gd-n** derivative compounds can act as an appropriate model to investigate the quantum coherence behaviors with tunable environmental influences. We successfully find the relationship between the molecule structures and quantum coherence behaviors and give suggestions on designing particular molecules for certain applications. By taking advantage of paramagnetic endohedral fullerenes, we believe such chemical engineering can enrich the interests in molecular design.

## Experimental

### Synthesis and isolation

The samples were synthesized and purified as described in our previous work.^23,24^

### EPR measurements

Gd@C_82_ and the three derivatives were dissolved in d_8_-toluene for EPR experiments with a spin concentration of 0.012 mmol L^−1^ determined by the spin counting method. The cw-EPR spectra were measured on a Bruker Elexsys E580 spectrometer with a superhigh sensitivity probehead (*ω* = 9.36 GHz). Pulsed EPR data were collected on the same system using an MS-3 cavity (*ω* = 9.33 GHz). The low-temperature environment was achieved using Oxford Instruments liquid helium cryostats (ESR900 for cw and CF935 for pulse). The cw-EPR spectra were simulated using an EasySpin^36^ toolbox (http://www.easyspin.org/) based on MATLAB. The signals of the pulsed-EPR experiments were collected by integrating the Hahn echo (π/2–*τ*–π–*τ*–echo) with *τ* = 200 ns. The *T*_1_ values were measured by the inversion recovery method (π–*T*–π/2–*τ*–π–*τ*–echo) with 4-step phase cycling. The *T*_M_ values were obtained by increasing the *τ* value of the Hahn echo sequence with 4-step phase cycling. The ESEEM measurements were carried out using the 2p ESEEM sequence (π/2–*τ*–π–*τ*–echo) with 4-step phase cycling. The dynamic decoupling measurements were carried out using the CPMG-*n* sequence (π/2_*x*_–(*τ*–π_*y*_–*τ*)_*n*_–echo) with 4-step phase cycling. The π/2 and π pulse lengths in EDFS, *T*_1_ and *T*_M_ measurements were 16 and 32 ns with 9 dB attenuation of the microwave power of 300 W, respectively.

### Magnetic measurements

The powder samples of **Gd-7** with exact mass for DC magnetic measurements were wrapped in parafilm and fixed in a capsule. DC experiments were performed on a Quantum Design MPMS XL-5 SQUID magnetometer. Magnetic data were corrected for the paramagnetism from the parafilm and capsule, and the diamagnetic contribution of the sample was calculated from Pascal's constants. The magnetization and magnetic susceptibility data were simulated using an EasySpin toolbox^36^ (http://www.easyspin.org/) based on MATLAB.

## Author contributions

Z. L. performed the EPR and magnetic measurements, assisted by B.-W. D. H. H. and B.-Y. S. prepared and purified the sample for measurements. Z. L. processed and analyzed the EPR and magnetic data. The project was conceived by S. G. and S.-D. J. Z. L. and S.-D. J. designed the experiments and wrote the manuscript. All the authors revised the manuscript.

## Conflicts of interest

The authors declare no conflict of interest.

## Supplementary Material

SC-011-D0SC02182B-s001
