# Supplementary material for: Amination of the Gd@C82 endohedral fullerene: tunable substitution effect on quantum coherence behaviors
Source: Chem Sci. 2020 May 28;11(39):10737–43. doi: 10.1039/d0sc02182b (PMC8162292; doi:10.1039/d0sc02182b)
Supplement: SC-011-D0SC02182B-s001 [file SC-011-D0SC02182B-s001.pdf]

Electronic Supplementary Material (ESI) for Chemical Science.
This journal is © The Royal Society of Chemistry 2020

Supporting Information

Amination of Gd@C_{82} endohedral fullerene: tunable substitution effect on quantum coherence behaviors

Zheng, Liu [a, †]; Huan, Huang [d, †]; Ye-Xin Wang [a]; Bo-Wei, Dong [a]; Bao-Yun, Sun [d *]; Shang-Da, Jiang [a, b *]; Song Gao [a, b, c *]

[a] Beijing National Laboratory of Molecular Science, Beijing Key Laboratory of Magnetoelectric Materials and Devices, College of Chemistry and Molecular Engineering, Peking University, Beijing 100871, China

[b] School of Chemistry and Chemical Engineering, South China University of Technology, Guangzhou 510640, China.

[c] Beijing Academy of Quantum Information Sciences, West Bld.#3,No.10 Xibeiwang East Rd., Haidian District, Beijing 100193, P. R. China

[d] CAS Key Lab for Biomedical Effects of Nanomaterials and Nanosafety, Institute of High Energy Physics, Chinese Academy of Sciences, Beijing 100049, China

[†] These authors contributed equally.

[*] Corresponding authors. E-mail:sunby@ihep.ac.cn; jiangsd@pku.edu.cn; gaosong@pku.edu.cn

List of Supporting Information

SI1 Electron paramagnetic resonance (EPR) measurements

SI2 Magnetic measurements for Gd@C_{82}(morpholine)$_7$

SI3 Quantum coherence properties measurements

SI1 Electron paramagnetic resonance (EPR) measurements

The Gd@C_{82} and the three analog compounds were dissolved in a d^8-toluene solution for EPR experiments with the spin concentration of 0.012 mmol/L determined by the spin counting method. Each sample was held into a 3-mm quartz EPR tube and froze and pumped for 5 times to exclude oxygen. The cw-EPR spectra were measured on a Bruker Elexsys E580 spectrometer with a superhigh sensitivity probehead (ω = 9.36 GHz). Pulsed EPR data were collected on the same system by an MS-3 cavity (ω = 9.33 GHz). The low-temperature environment was achieved by Oxford Instruments liquid helium cryostats (ESR900 for cw and CF935 for pulse). The cw-EPR spectra were simulated by EasySpin toolbox based on MATLAB. The signal of the pulsed-EPR experiments were collected by integrating the Hahn echo (π/2-τ-π-τ-echo) with τ = 200 ns. The T_1 values were measured by the inversion recovery method (π-T-π/2-τ-π-τ-echo) with 4-step phase cycling. The T_M values were obtained by increasing the τ value of the Hahn echo sequence with 4-step phase cycling. The ESEEM measurements were carried out by the 2-pulse-ESEEM sequence (π/2-τ-π-τ-echo) with 4-step phase cycling. The dynamic decoupling measurements were carried out by the CPMG sequence ($\pi/2_x$-$(\tau$-π_y-$\tau)_n$-echo) with 4-step phase cycling. The π/2 and π pulse lengths in EDFS, T_1 and T_M measurements, were 16 and 32 ns with 9 dB attenuation of the microwave power of 300 W, respectively.

SI2 Magnetic measurements for Gd@C_{82}(morpholine)$_7$

Gd-7 is considered as ***S*** = 7/2 in cw-EPR measurements with ***g*** = 1.98. The pure sample of **Gd-7** in toluene solution was dried under vacuum for 24 hours. Then the powder sample (0.67 mg) was move onto parafilm (5.03 mg) and fixed in the capsule. DC experiments were performed on a Quantum Design MPMS XL-5 SQUID magnetometer. Magnetic data were corrected for the paramagnetism from the parafilm and capsule, and the diamagnetic contribution of the sample was calculated from Pascal's constants. All magnetic data were collected with the applied magnetic field from 0 to 5 T at 2 K. The saturation magnetization for **Gd-7** gives an effective magnetic moment of 6.77 μ_B per molecule. The theoretical saturation magnetization can be calculated using the following formula

$M_S = NgS\mu_B$#(2.1)

where M_s represents the saturation magnetization, N for the spin concentration, g for the g-factor, S for the spin quantum number and μ_B for the Bohr magneton. The experimental value fit with the ***S*** = 7/2 situation as shown in Table S2-1.

Table S2-1. The theoretical value for saturation magnetization.

S	3	7/2	4
theoretical value	5.8661 μ_B	6.8561 μ_B	7.8461 μ_B

Theoretical curves for ***S*** = 3, 7/2 and 4 were calculated using Brillouin function as the following description

$M = M_s B_s(x)$,#(2.2)

$B_s(x) = \frac{2S+1}{2S}\coth\left(\frac{2S+1}{2S}x\right) - \frac{1}{2S}\coth\left(\frac{1}{2S}x\right)$,#(2.3)

$x = \frac{gS\mu_B H}{k_B T}$,#(2.4)

where k_B represent the Boltzmann constant. The spin effective magnetic moment values for experimental points and theoretical curves are shown in Figure S2-1.

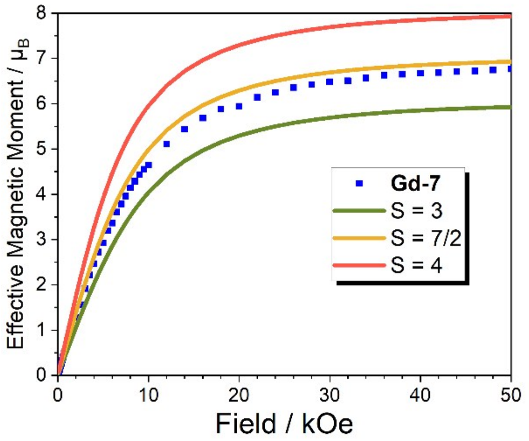

Figure S2-1. The spin effective magnetic moment values for experimental points and theoretical curves. The experimental data is shows as blue point. Three theoretical calculations for ***S*** = 3, 7/2 and 4 are shown as colored curves.

SI3 Quantum coherence properties measurements

3.1 Spin-lattice relaxation (T_1) at different temperatures

T_1 were measured using inverse-recovery method. The pulse sequence is π-T-π/2-τ-π-τ-echo with fixed τ and varied T. We used π/2 = 16 ns and π = 32 ns pulse for the measurements. τ = 200 ns was chosen for detection. 4-step phase cycling was used to eliminate the contribution of unwanted free-induction decay (FID) and echo signals. The experiment data are shown in Table S3-1.

Table S3-1. The T_1 for Gd@C_{82} and the three analog compounds at different temperature. Unlisted experimental values are not available due to the weak spin echo signal.

Temperature	**Gd@C_{82}**	**Gd-5**	**Gd-7**	**Gd-9**
5 K	1.70(1) μs	62.1(5) μs	71.7(2) μs	103.7(2) μs
7 K	1.02(1) μs	16.2(1) μs	25.8(3) μs	24.5(4) μs
10 K	0.59(1) μs	6.35(4) μs	8.88(9) μs	10.9(1) μs
15 K		2.10(1) μs	2.60(3) μs	4.61(8) μs
20 K		1.10(1) μs	1.03(3) μs	1.21(3) μs
25 K		0.80(1) μs		1.57(1) μs
30 K		0.61(3) μs		1.13(1) μs
40 K		0.50(1) μs		0.80(1) μs

3.2 Quantum Phase Memory Time (T_M) at different temperatures

T_M were measured using Hahn-echo decay method. The pulse sequence is π/2-τ-π-τ-echo with τ varies. We used π/2 = 16 ns and π = 32 ns pulse for the measurements. τ = 200 ns was chosen for initial interval. 4-step phase cycling was used to eliminate the contribution of unwanted free-induction decay (FID) and echo signals. The experiment data are shown in Table S3-2.

Table S3-2. The T_M for Gd@C_{82} and the three analog compounds at different temperature. Unlisted experimental values are not available due to the weak spin echo signal.

Temperature	**Gd@C_{82}**	**Gd-5**	**Gd-7**	**Gd-9**
5 K	0.564(5) μs	5.1(6) μs	4.4(8) μs	4.5(3) μs
7 K	0.408(2) μs	3.7(5) μs	3.7(6) μs	4.3(9) μs
10 K	0.244(3) μs	3.1(3) μs	2.9(4) μs	2.9(3) μs
15 K	0.24(3) μs	1.8(2) μs	1.6(2) μs	1.9(1) μs

3.3 Enhanced T_M using dynamic decoupling

Enhanced T_M were measured using Hahn-echo decay method. The pulse sequence is $\pi/2_x$-$(\tau$-π_y-$\tau)_n$-echo with τ variation. We used π/2 = 16 ns and π = 32 ns pulse for the measurements. τ = 200 ns was chosen for initial interval. 4-step phase cycling was used to eliminate the contribution of unwanted free-induction decay (FID) and echo signals. The experiment data are shown in Table S3-3.

Table S3-3. The enhanced T_M for the **Gd-n** using different number of inversion pulse number. Unlisted experimental values are not available due to the weak spin echo signal.

Inversion pulse number	**Gd-5**	**Gd-7**	**Gd-9**
1	5.1(6) µs	4.4(8) µs	4.5(3) µs
2	5.6(5) µs	6.4(7) µs	6.9(6) µs
4	7.1(7) µs	8.2(9) µs	7.8(7) µs
8	9.7(8) µs	10.6(4) µs	10.2(9) µs
16		15(3) µs	14(4) µs
